# Cystatin C as a Predictor of Mortality and Cardiovascular Events in a Population with Chronic Kidney Disease

**DOI:** 10.1155/2014/127943

**Published:** 2014-02-11

**Authors:** Ana Vigil, Emilia Condés, Luis Vigil, Paloma Gallar, Aniana Oliet, Olimpia Ortega, Isabel Rodriguez, Milagros Ortiz, Juan Carlos Herrero, Carmen Mon, Gabriela Cobo, Juana Jimenez

**Affiliations:** ^1^Department of Nephrology, Nephrology Service, Hospital Universitario Severo Ochoa, Avenida. Orellana s/n, Leganés, 28911 Madrid, Spain; ^2^Department of Medical Specialties, Psychology and Applied Pedagogy, Universidad European de Madrid, Villaviciosa de Odon, 28670 Madrid, Spain; ^3^Hypertension Unit, Department of Internal Medicine, Hospital Universitario de Mostoles, Móstoles, 28935 Madrid, Spain; ^4^Department of Biochemistry, Hospital Universitario Severo Ochoa, Leganes, 28911 Madrid, Spain

## Abstract

*Background*. We examine whether cystatin C, a surrogate marker of renal function, could identify patients with chronic kidney disease (CKD) with an increased risk of renal disease progression, death, or cardiovascular events. *Methods*. Data were obtained for 180 patients, with a diagnosis of chronic renal failure based on serum creatinine estimated glomerular filtration rate (eGFR_creat_) <90 mL/min/1.73 m^2^. This population was grouped in tertiles according to cystatin C and creatinine values at baseline. Cardiovascular events and overall mortality were estimated for each tertile. Predictors of overall mortality and for the development of renal disease progression were analyzed. *Results*. The median age was 75 years (interquartile range 69–82) and the median eGFR_creat_ 38 mL/min m^2^ (interquartile range 33–49). Overall mortality was lower on the first and on the second tertiles of cystatin C than on the third one (HR = 0.060; 95% CI: 0.008–0.447 and HR = 0.094; 95% CI: 0.022–0.406, resp.). Deaths related to the creatinine tertiles followed the same pattern, but differences were not as large. Cardiovascular mortality was lower on the second than on the third cystatin C tertile (HR = 0.198; 95% CI: 0.040–0.987), but it did not show differences on the first and the second creatinine tertiles compared with the third one (HR = 0.126; 95% CI: 0.013–1.265 and HR = 0.403; 95% CI: 0.093–1.740). The only independent predictors of mortality during followup were baseline cystatin C (OR = 0.100; 95% CI: 0.021–0.463) and baseline uric acid (OR = 1.377; 95% CI: 1.070–1.773). *Conclusion*. Cystatin C may be an alternative to creatinine for detecting a high risk of death and cardiovascular events in a population with CKD.

## 1. Introduction

Chronic kidney disease is a worldwide health problem that carries a significant risk of cardiovascular morbidity and mortality.

Endogenous filtration markers have been used as tests of kidney function, with serum creatinine as the most widely applied marker. Estimated glomerular filtration rate (eGFR) based on serum creatinine (eGFR_creat_) does not fully account for non-GFR determinants of creatinine (muscle mass, race, age, and gender).

An alternative endogenous serum biomarker, cystatin C, has been proposed for estimating renal function that can replace or supplement serum creatinine. In multiple studies it has been shown to be more sensitive for predicting adverse events than serum creatinine or eGFR_creat_. This parameter also showed greater sensitivity to detect mild reductions in renal function and improved the identification of patients with higher cardiovascular risk in epidemiological studies [1–3]. The association of cystatin C with metabolic syndrome and classic cardiovascular risk factors is also well documented [4–6]. This association may reflect the inflammatory components of the syndrome. A positive correlation between cystatin C and inflammation parameters including interleukin-6, resistin, tumor necrosis factor, and C-reactive protein has been reported [6]. Cystatin C is emerging as a new biomarker in cardiovascular disease [7].

All the aforementioned may suggest that cystatin C could be more useful for predicting adverse clinical events and become a clinical tool to optimize the estimation of glomerular filtration rate [3].

The purpose of our study was to analyze whether cystatin C and eGFR formulas based on cystatin C (eGFR_cyst_) identify a subgroup of patients with an increased risk of progression of renal failure, cardiovascular events, and overall mortality among a group of selected patients, improving the standard method of creatinine (eGFR_creat_) for the diagnosis and followup of renal failure.

## 2. Subjects and Methods 

We conducted a longitudinal, observational, and retrospective cohort study of a sample extracted from 589 patients referred to the Nephrology clinic between June 2005 and May 2011, derived from Primary Care with the diagnosis of renal failure, defined by a glomerular filtration formula estimated through eGFR_creat_ < 90 mL/min/m² and confirmed in a second determination in a 3-month period. Those who had at least a cystatin C determination in this period were selected. Those with thyroid dysfunction or inflammatory pathology or receiving steroid treatment, factors all known to alter the concentration of serum cystatin C, were excluded and only those that had a minimum followup of 3 months were included. At the end, 180 patients were selected. The Nephrology Clinic covers the Health Care Area at the town of Leganes, a suburb near Madrid, with a population of 187,227 inhabitants registered during the study period.

Cardiovascular events (heart failure, acute myocardial infarction, and stroke) and mortality for both cardiovascular events and other causes were registered during followup. An acute myocardial infarction was diagnosed when there was evidence of myocardial necrosis in association with clinical signs of myocardial ischemia. Necrosis was diagnosed on the basis of a rising or falling pattern of the local cardiac troponin level. Stroke (ischemic or hemorrhagic) was defined as an acute reduction of cerebral blood flow causing transient or permanent loss of neurologic function. An acute decompensated heart failure was diagnosed on the basis of the presence of at least one symptom (dyspnea, orthopnea, or edema) and one sign (rales, peripheral edema, ascites, or pulmonary vascular congestion on chest radiography) of heart failure.

Death was documented in the medical report released to the Admission Service. Therefore, patients who could die at home or in other Center were not registered. Cardiovascular events were documented from the medical reports used during hospitalization and/or the emergency services. Events occurring in other centers were included only when a medical report of the corresponding center recorded the fact. A renal event was defined as the development of eGFR_creat_ ≤ 20 mL/min/1.73 m^2^ during the follow-up.

### 2.1. Analytical Methods

The serum concentration of cystatin C was measured using nephelometry BNII, Siemens. Albuminuria determination was conducted in first morning voided urine using the albumin/creatinine ratio. In cases of albuminuria values > 400 mg/gr creatinine, proteinuria determination was performed in a 24 h urine collection.

Glomerular filtration rate (GFR) at baseline and during followup was estimated by the following equations:

(1) eGFR-EPI-cyst=127.7×cyst−1.17×age−0.13 ×(0.91  if  female)×(1.06  if  black)

(see [8]),

(2) eGFR-EPI-creat=141×min⁡(SCr/κ,1)α ×max⁡(SCr/κ,1)−1.209×0.993age ×1018(if  female)×1.159(if  black),

where SCr is serum creatinine in mg/dL, *κ* is 0.7 for females and 0.9 for males, *α* is −0.329 for females and −0.144 for males, min indicates the minimum of SCr/*κ* or 1, and max indicates the maximum of  SCr/*κ* or 1 [9].

### 2.2. Statistical Analysis

In the descriptive study results are expressed as mean and standard deviation or median and interquartile range for continuous variables depending on whether they followed or not a normal distribution. Qualitative variables were expressed as absolute and relative frequencies. Variables cystatin C and creatinine were grouped into tertiles. We calculated the incidence rate of cardiovascular events and mortality for these tertiles. Cox regression analysis was performed to calculate the risk of events, adjusted for age, sex, BMI, previous cardiovascular events and tobacco consumption. For the contrasts, univariate analysis of variance or Kruskal-Wallis test or logistic regression was performed.

For multivariate analysis we used test of binary logistic regression adjusted for age, sex, BMI, previous cardiovascular events and tobacco consumption. The selection of variables was performed by the Wald method. Confidence intervals were calculated at 95%. The level of statistical significance was *α* < 0.05. All analysis was performed using SPSS version 15.0 (SPSS Inc., Chicago, IL).

The primary end point was to analyze the risk of overall mortality in relation to renal function. As secondary end point we analyze the development of cardiovascular events (fatal and nonfatal) or entry on dialysis for ESRD.

## 3. Results

### 3.1. Baseline Characteristics of the Study Population

The total number of patients was 180 (52% females). Median age was 75 (69–82) years and mean followup was 36.55 ± 15.98 months.  Table 1 describes the baseline characteristics of the study sample by tertile's category of cystatin C and serum creatinine at baseline.

Patients with higher levels of cystatin C (third tertile) were older and had higher levels of creatinine and lower eGFR_creat_ and eGFR_cyst_. Baseline cholesterol was lower in patients with higher cystatin C levels. Patients with higher levels of creatinine (third tertile) were predominantly male and had higher cystatin C while eGFR_creat_ and eGFR_cyst_ were lower. The rest of the variables analyzed showed no differences in any of the two categories.

### 3.2. Incidence of Cardiovascular Events and Overall Mortality

Total followup of the study was 525 persons/year. The incidence of global cardiovascular events was 306/1000 person-years (Table 2) without differences between the patients with higher levels of cystatin C or creatinine. Nonfatal cardiovascular events (235/1000 person-years) showed neither difference by category neither of cystatin C nor of creatinine.

Compared with the third tertile, patients on the second tertile of cystatin C had a lower risk of fatal cardiovascular event (HR = 0.198, 95% CI: 0.040–0.987). Global mortality was also lower for the first cystatin C tertile (HR = 0.060, 95% CI: 0.008–0.447) and for the second tertile (HR = 0.094, 95% CI: 0.022–0.406) with respect to the third tertile. For the tertiles of creatinine we also found a lower risk in the first and the second than in the third (HR = 0.178, 95% CI: 0.039–0.805 and HR = 0.329, 95% CI: 0.115–0.442, resp.).

Causes of death were cardiovascular events in 12 patients, infectious cause in 7 patients, and tumor in 1 patient. A patient died in end-stage renal disease (ESRD) after rejecting dialysis.

There were 11 renal events that were less frequent in patients with lower levels of creatinine (HR = 0.142, 95% CI: 0.035–0.577). A lower level of cystatin C was associated with a reduced incidence of renal events, although without showing differences among tertiles. Entry on dialysis only occurred in two patients during the follow-up.

### 3.3. Renal Function at Baseline

Mean eGFR_creat_ value at the beginning of the study was 38 (33–49) mL/min/1.73 m^2^. Their distribution was 19 patients (11%) in stage 2, 137 patients (75.7%) in stage 3, and 24 (13.2%) in stage 4. The medium eGFR_cyst_ at the beginning of the study was 41 (32–52) mL/min/1.73 m^2^; 27 patients (15.5%) were in stage 2, 118 patients (65.2%) in stage 3, and 35 patients (19.3%) in stage 4 according to NKF KDOQI classification [8].

**Figure 1:**
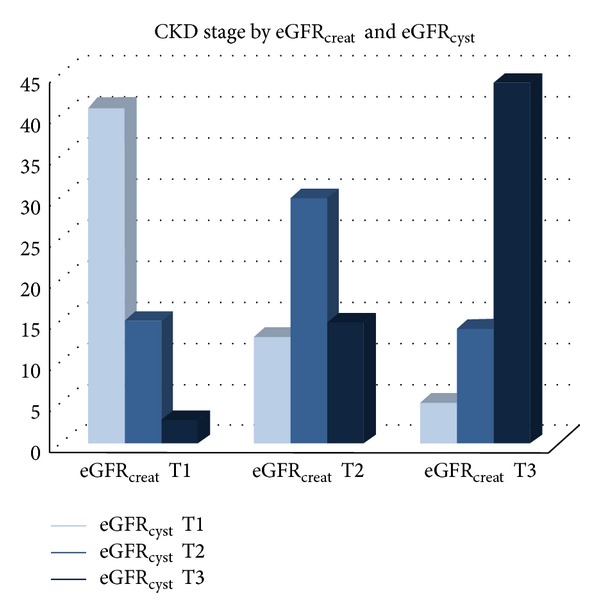
Distribution of patients according to estimated GFR defined by cystatin C and creatinine tertiles. CKD: chronic kidney disease; eGFR_creat_ and eGFR_cyst_: estimated glomerular filtration rate according to creatinine and cystatin C; T1: tertile 1; T2: tertile 2; T3: tertile 3.

When comparing the stage of renal failure between eGFR_creat_ and eGFR_cyst_, for a matching on the estimated stage of kidney failure, we found a concordance in 63.88% of patients (*n* = 115). Discordance of both took place in 17.7% (*n* = 32 patients) with eGFR_creat_ < eGFR_cyst_ and 18.3% (*n* = 33 patients) with eGFR_cyst_ < eGFR_creat_ (Figure 1).

### 3.4. Multivariate Analysis

**Figure 2:**
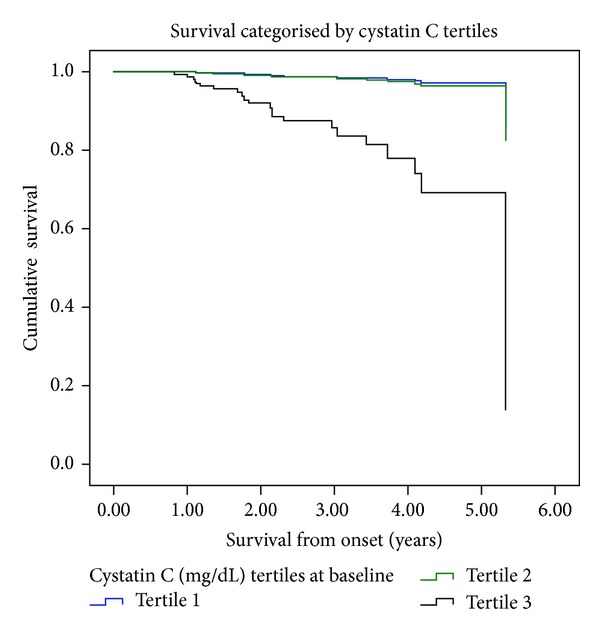
Survival and tertiles of cystatin C.

**Figure 3:**
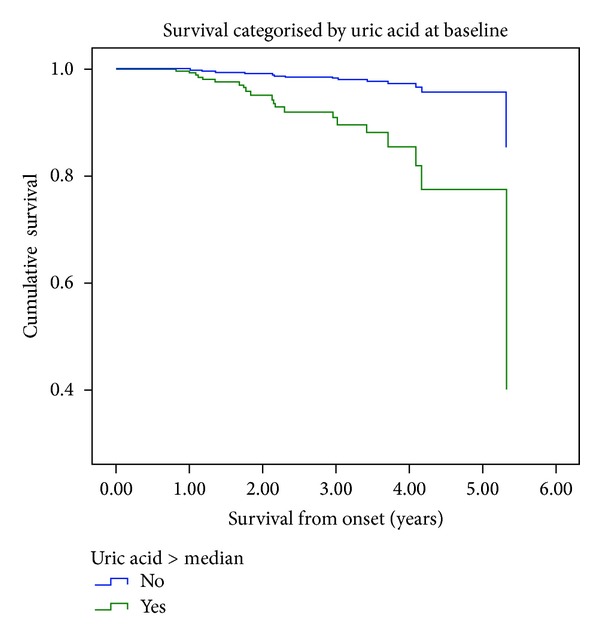
Survival and baseline uric acid levels.

Cystatin C categorized into tertiles and baseline uric acid levels were the only independents predictors of overall mortality, adjusted for age, sex, BMI, tobacco consumption, and a history of a previous cardiovascular event (Table 3, Figures 2 and 3).

## 4. Discussion

In our study death was more frequent than the progression to ESRD. Unlike creatinine, basal serum cystatin C was a predictor of overall mortality and of the development of fatal cardiovascular events. We also found that basal serum uric acid was an independent predictor of overall mortality along with cystatin C.

The higher incidence of exitus versus initiation of dialysis has also been described in at least two other previous studies. In one of them analyzing the natural history of CKD in a population of 27,998 patients in the USA, with an estimated GFR < 90 mL/min/1.73 m², Keith et al. [10] found that the incidence of dialysis treatment during a 5-year follow-up was 1.1%, 1.3%, and 19.9% for stages 2, 3, and 4, respectively, of the classification renal K/DOQI. However, mortality was 19.5%, 24.3%, and 45.7%. The authors conclude that death was more frequent than entering on dialysis at all stages of kidney failure. In the second observation, Go et al. [11] found an independent association between eGFR_creat_ < 60/mL/min/m² and the risk of death or hospitalization for cardiovascular events in a cohort of 1,120,295 adults in a community followed an average of 2.8 years.

The patients in our series were selected by their renal insufficiency, with 88% of them in stage ≥3 as classified by K/DOQI [12]. They as well had a higher mean age and 39% exhibit a type 2 diabetes mellitus diagnosis. As we only considered deaths occurring at our center (either at the emergency room or during hospitalization) it is likely that we underestimated total mortality since we can not rule out that some deaths occurred at home or in other hospital. These results suggest that the population finally developing ESRD could be considered as a specific group of patients surviving other causes of death and thus reaching the point of chronic dialysis.

The second finding of our study was that cystatin C levels were a strong independent predictor of overall mortality and cardiovascular mortality. Patients with lower levels of cystatin C had an incidence of fatal cardiovascular events and overall mortality significantly lower compared with the higher, something not happening with creatinine levels.

These results are consistent with previous studies. Shlipak et al. [13] using a cohort of 4663 participants in the Cardiovascular Health Study (CHS) [14, 15] recruited from four U.S. communities studied cystatin C as a prognostic biomarker of death, cardiovascular disease, and incidental chronic kidney disease in people > 65 years without previous renal disease. They conclude that serum cystatin C was a better predictor than creatinine for the development of the mentioned events and identifies a state of “preclinical” renal dysfunction with cystatin C, which would not be detected by eGFR_creat_ alone. Using data from the MESA and CHS study, Peralta et al. [16] found that the eGFR_cyst_ predicts death and cardiovascular complications better than eGFR_creat_ and identifies nondiabetic patients with CKD stage 3 not detected by eGFR_creat_, with an increased risk of complications.

In our population hyperuricemia, along with cystatin C, was as well an independent predictor of overall mortality. A large number of epidemiological studies have suggested the independent role of hyperuricemia in overall mortality, cardiovascular disease, and kidney disease in the general population [17–21]. However, in patients with this condition, it is less clear whether uric acid is just a marker that reflects a set of comorbidities and kidney damage or a true causal risk factor. Three previous works [22–24] studied the association between uric acid and mortality in patients with chronic renal failure.

Concerning the follow-up of renal function we have only analyzed the deterioration of renal function, defined as renal event, and the low number of patients who progressed to ESRD and dialysis prevented us from performing a statistical analysis of this variable. The incidence of renal events was only related to higher levels of basal serum creatinine and not to basal cystatin C levels. When comparing renal function estimated by the two markers (creatinine and cystatin C) concordance and discordance in the stage of renal failure was similar to that found by other authors. Krolewski et al. [25], in a study of two cohorts of diabetic patients, concluded that renal function estimated with cystatin C significantly improves the prediction of the risk of progression to ESRD compared to the estimate achieved with creatinine. Our results allow us to venture that with a greater number of patients and higher renal events our findings would probably be similar to their work.

Being retrospective, our study has several limitations. Only patients with a determination of cystatin C, 36.16% of transferred patients, were included. Of these, 84.5% had renal failure stage ≥ 2. We ignore the criteria for this first cystatin C determination in each case, although it is logical to assume that it was performed as a parameter of renal function in addition to serum creatinine.

The insufficient number of patients with progression to ESRD prevented us from performing a statistical study of one of our goals: to check whether cystatin C was a more sensitive marker than creatinine for predicting the development of ESRD. However, our findings about the predictive value of hyperuricemia and cystatin C for the development of fatal cardiovascular events and global mortality in a referral population at a high risk for progression of renal disease have, in our opinion, clinical relevance. Our study, as those of other authors [26], supports the use of a combination of markers to improve the detection and risk stratification of patients with high cardiovascular risk and chronic renal failure.

The follow-up time was relatively short (average 3 years) but the high mean age of our patients could make up for this draw back. Another weakness stems also from the retrospective nature of the study: some of the analyzed events were probably developed at other centers and were not included. Thus, death rate may be underestimated (only those occurring at our center were considered). Despite this, the number of total events seems to us sufficient to draw conclusions with enough statistical power.

In conclusion, our study found that during a follow-up period of 3 years, cystatin C and hyperuricemia were the only independent predictors of total mortality. Unlike creatinine cystatin C was predictive of fatal cardiovascular events. These findings support the usefulness of including uric acid and cystatin C as markers for the assessment of cardiovascular risk morbidity and mortality in patients with chronic renal failure.

## Figures and Tables

**Table 1:** Baseline characteristics of the study sample by tertile's category of cystatin C and serum creatinine.

	Total *N* = 180	Cystatin C			*P* value	Creatinine			*P* value
		Tertile 1 *N* = 59	Tertile 2 *N* = 60	Tertile 3 *N* = 61		Tertile 1 *N* = 59	Tertile 2 *N* = 56	Tertile 3 *N* = 65	
Age (years)*	75 (69–82)	70 (65–78)	74 (70–80)	81 (74–83)	<0.001	75 (67–80)	76 (71–82)	74 (69–82)	0.362
Gender (female%)	95 (52)	36 (61)	32 (53)	27 (44)	0.744	32 (54)	36 (64)	17 (26)	<0.001
SBP (mm Hg)*	140 (130–150)	140 (130–150)	140 (130–150)	140 (125–150)	0.006	140 (130–150)	140 (130–150)	140 (126–160)	0.770
DBP (mm Hg)*	80 (70–80)	80 (70–80)	80 (71–89)	70 (62–80)	0.004	80 (75–80)	80 (70–80)	80 (68–80)	0.150
BMI (Kg/m^2^)*	29 (26–33)	28.7 (26.8–32.6)	31.4 (27.2–33.6)	27.1 (23.9–32.2)	0.946	29.6 (26.5–32.9)	30.5 (26.7–33.9)	27.9 (24.6–32.0)	0.041
Proteinuria (gr/24 h)*	0.14 (0.08–0.27)	0.14 (0.08–0.27)	0.13 (0.08–0.32)	0.15 (0.08–0.29)	0.092	0.13 (0.08–0.22)	0.13 (0.05–0.28)	0.15 (0.08–0.32)	0.318
Glucose (mg/dL)*	102 (93–128)	106 (94–129)	104 (94–140)	98 (90–120)	0.006	103 (93–122)	111 (94–143)	100 (91–115)	0.058
Serum uric acid (mg/dL)*	6.9 (5.8–8.1)	6.5 (5.7–7.4)	6.9 (5.9–7.9)	7.5 (6.1–9.8)	0.087	6.4 (5.5–7.4)	7.0 (5.9–8.4)	7.4 (6.2–9.4)	0.004
Total cholesterol (mg/dL)*	185 (154–212)	188 (165–219)	182 (158–215)	175 (148–201)	<0.001	197 (168–225)	177 (151–201)	181 (151–202)	0.026
Serum creatinine (mg/dL)*	1.5 (1.3–1.8)	1.3 (1.2–1.5)	1.6 (1.4–1.8)	1.7 (1.4–2.1)	<0.001	1.2 (1.1–1.3)	1.5 (1.4–1.6)	1.8 (1.7–2.0)	<0.001
eGFR_creat_ (mL/min/1.73 m^2^)*	38 (33–49)	52 (42–66)	38 (34–44)	32 (28–36)	<0.001	56 (44–66)	36 (32–43)	33 (28–37)	<0.001
eGFR_cyst_ (mL/min/1.73 m^2^)*	41 (32–52)	59 (52–67)	41 (39–44)	28 (24–32)	<0.001	54 (42–66)	39 (29–48)	36 (26–42)	<0.001
Serum cystatin C (mg/dL)*	1.6 (1.3–1.9)	1.2 (1.1–1.3)	1.6 (1.5–1.7)	2.1 (1.9–2.5)	<0.001	1.3 (1.1–1.5)	1.6 (1.4–2.0)	1.8 (1.6–2.4)	<0.001
Diabetes mellitus, *n* (%)	70 (39)	19 (32)	25 (42)	26 (43)	0.436	19 (32)	28 (50)	23 (35)	0.113
Tobacco, *n* (%)	74 (41)	30 (51)	21 (35)	23 (38)	0.171	21 (36)	15 (27)	38 (58)	0.001
ACEI, *n* (%)	88 (49)	30 (51)	28 (47)	30 (49)	0.900	27 (46)	31 (55)	30 (46)	0.506
ARB, *n* (%)	47 (26)	19 (33)	15 (25)	30 (49)	0.386	16 (27)	17 (30)	14 (21)	0.533
Hypolipidemics treatment *n* (%)	80 (44)	19 (33)	25 (42)	25 (41)	0.481	26 (44)	24 (43)	30 (46)	0.934
Previous CV event (%)	68 (38)	17 (29)	21 (35)	30 (49)	0.070	13 (22)	27 (48)	28 (44)	0.008

SBP: systolic blood pressure; DBP: diastolic blood pressure; BMI: body mass index; eGFR-EPI: estimated glomerular filtration rate according to creatinine (eGFR_creat_) and according to cystatin C (eGFR_cyst_); *n*: number; CV: cardiovascular; ACEI: angiotensin converting enzyme inhibitor; ARB: angiotensin receptor blocker. *Data expressed as median and interquartil range.

**Table 2:** Incidence of cardiovascular events and overall mortality by cystatin C and serum creatinine, categorized by tertiles.

	Cystatin C			Creatinine		
	Tertile 1	Tertile 2	Tertile 3	Tertile 1	Tertile 2	Tertile 3
Participants number	59	60	61	59	56	65
Persons/year	166	196	163	198	151	177
Total cardiovascular events						
Participants number	13	17	23	16	15	22
Incidence/1000 persons-year	78	87	141	81	99	124
HR	0.782 (0.363–1.688)	0.743 (0.381–1.449)	—	0.802 (0.401–1.602)	0.715 (0.345–1.478)	—
Fatal cardiovascular events						
Participants number	0	2	10	1	4	7
Incidence/1000 persons-year	0	10	61	5	26	39
HR	0	**0.198 (0.040–0.987)**	—	0.126 (0.013–1.265)	0.403 (0.093–1.740)	—
Non-fatal cardiovascular events						
Participants number	13	15	13	15	11	15
Incidence/1000 persons-year	78	77	80	76	73	85
Total mortality						
Persons-year	166	199	176	198	151	192
Participants number	1	2	18	2	5	14
Incidence/1000 persons-year	6	10	102	10	33	73
HR	**0.060 (0.008–0.447)**	**0.094 (0.022–0.406)**	—	**0.178 (0.039–0.805)**	**0.329 (0.115–0.442)**	—
Renal events						
Persons-year	166	197	166	198	151	177
Participants number	0	5	6	0	2	9
Incidence/1000 persons-year	0	25	36	0	13	51
HR	0	0.463 (0.095–2.254)	—	0	**0.142 (0.035–0.577)**	—

Event risks were evaluated in Cox proportional model, adjusted for age, gender, BMI, previous cardiovascular event, and tobacco consumption. Values in bold letters means that  *P* < 0.005; HR: hazard risk.

**Table 3:** Estimation of total mortality and renal event risk.

Event	Parameters		O.R.	95% CI	*P* value
Total mortality	Uric acid levels		1.377	(1.070–1.773)	0.013
	Cystatin C	Tertile 1	0.062	(0.008–0.497)	0.009
		Tertile 2	0.100	(0.021–0.463)	0.003

Renal events	Creatinine	Tertile 1	0	—	—
		Tertile 2	0.156	(0.043–0.568)	0.005

Logistic regression analysis of total mortality and renal events, adjusted by age, gender, uric acid, and tobacco consumption.
